# Besonderheiten der Risiko- und Krisenkommunikation im Strahlenschutz und radiologischen Notfallschutz

**DOI:** 10.1007/s00103-022-03525-y

**Published:** 2022-04-12

**Authors:** Christiane Pölzl-Viol

**Affiliations:** DO 4 Risikokommunikation und Wissensmanagement, Bundesamt für Strahlenschutz, Ingolstädter Landstr. 1, 85764 Oberschleißheim-Neuherberg, Deutschland

**Keywords:** Risikokommunikation, Krisenkommunikation, Risikowahrnehmung, Strahlenschutz, Radiologischer Notfallschutz, Risk communication, Crisis communication, Risk perception, Radiation protection, Radiological emergency management

## Abstract

Die Gestaltung einer guten Risiko- und Krisenkommunikation im Strahlenschutz ist stets in Bezug zum gesellschaftlichen Umgang mit den verschiedenen Strahlenthemen zu sehen, zu denen die Kommunikation stattfindet. Risiko- und Krisenkommunikation werden dabei meist als unterschiedliche Kommunikationsdisziplinen betrachtet. Der Artikel gibt einen Einblick in die Komplexität der Kommunikation im Strahlenschutz in unterschiedlichen Kontexten. Er beschreibt die Einsatzmöglichkeiten der jeweiligen Kommunikationsform und die Zusammenhänge zwischen diesen Kommunikationsformen.

## Abstract (en)

Forms of good risk and crisis communication in radiation protection must always be seen in relation to the societal handling of the different radiation topics about which the communication takes place. Risk and crisis communication are usually regarded as different communication disciplines. The article provides insight into the complexity of communication in the field of radiation protection in different contexts. Special characteristics of the respective form of communication and the possible uses of these forms of communication are described.

## Einleitung

Spätestens nach dem Reaktorunfall von Tschernobyl im Jahr 1986 wurde deutlich, dass Krisen- und Risikokommunikation für den Strahlenschutz unerlässlich sind und Strahlung ein besonders anspruchsvolles Feld für die Kommunikation ist. Strahlung ist ein klassischer Angstauslöser, weil man sie nicht sehen, nicht riechen und nicht schmecken kann und mögliche Wirkungen auf die Gesundheit in der Regel verzögert und lang anhaltend auftreten. Dies und die historischen Bilder von den Opfern der Atombombenabwürfe auf Hiroshima und Nagasaki im Jahr 1945 sowie die Berichterstattung über die Atomreaktorkatastrophen von Tschernobyl und Fukushima (im Jahr 2011) erhöhen die Angst vor Kontamination oder Erkrankung [1]. Aber auch Themen aus anderen Bereichen des Strahlenschutzes, wie z. B. der Mobilfunk- und Stromnetzausbau, besitzen in Teilen der Bevölkerung eine besonders hohe Aufmerksamkeit und bergen Herausforderungen für die Risikokommunikation.

Die Ziele und Aufgaben der Risiko- und Krisenkommunikation werden häufig getrennt voneinander betrachtet. Allgemein wird der Begriff Risikokommunikation für Themen verwendet, in denen es unabhängig von der Frage, ob es jemals zu einer Krisensituation kommen kann, darum geht, ein Risiko besser zu erklären und zielgruppenspezifisch, rezipientenorientiert und wirkungsvoll mit der Öffentlichkeit zu kommunizieren. Definitionen von Risikokommunikation sprechen von einem „Austausch von Informationen und Meinungen über Risiken, zur Risikovermeidung, -minimierung und -akzeptanz“ [2]. Ein wichtiges Ziel der Risikokommunikation ist der Aufbau eines Vertrauensverhältnisses zwischen staatlichen Stellen und den Bürger*innen bzw. deren Repräsentanten [3]. Krisenkommunikation hingegen ist Kommunikation in einer Krisensituation und Bestandteil des Katastrophenmanagements. Sie soll durch schnelle und angemessene Reaktion dazu beitragen, die physische und psychische Gesundheit der betroffenen Bevölkerung im Krisenfall zu erhalten [4].

Ziel des folgenden Beitrags ist es, anhand der Besonderheiten der Risiko- und Krisenkommunikation im Strahlenschutz und anhand des gesellschaftlichen Umgangs mit Strahlenthemen zu diskutieren, inwieweit Risiko- und Krisenkommunikation zeitlich und thematisch voneinander getrennt zu betrachten sind und inwieweit eine gelungene Risikokommunikation im Vorfeld unabdingbare Voraussetzung für eine gute Krisenkommunikation ist. Dabei wird zunächst auf die Diversität der Risikokommunikation zu verschiedenen Strahlenthemen eingegangen, gefolgt von den Herausforderungen für die Krisenkommunikation in einem radiologischen Notfall. Die Bedeutung von Risiko- und Krisenkommunikation hierbei wird beschrieben. Abschließend wird auf die Abgrenzung und die Gemeinsamkeiten der beiden Kommunikationsformen eingegangen.

## Diversität der Risikokommunikation im Strahlenschutz

### Zuständigkeiten im Strahlenschutz und radiologischen Notfallschutz

Das Bundesamt für Strahlenschutz (BfS) ist in Deutschland die für den Strahlenschutz zuständige Bundesoberbehörde im Geschäftsbereich des Bundesministeriums für Umwelt, Naturschutz, nukleare Sicherheit und Verbraucherschutz (BMUV). Das BfS hat ein breites Spektrum an Aufgaben in den Bereichen Umwelt und Gesundheit, Wirkungen und Risiken von ionisierender und nichtionisierender Strahlung, radiologischer Notfallschutz, Überwachung der Umweltradioaktivität sowie medizinischer und beruflicher Strahlenschutz.^1^ Die Information und Kommunikation über Strahlenrisiken und die Kommunikation in einem radiologischen Notfall sind wichtige Aufgaben des BfS. Auch in den Bundesländern sind Behörden für den Strahlenschutz und den radiologischen Notfallschutz angesiedelt. Zusätzlich übernehmen die Katastrophenschutzbehörden die entsprechenden Aufgaben in einem radiologischen Notfall. Die nachfolgenden Beschreibungen erfolgen aus Sicht des BfS.

Risikokommunikation spielt in allen Strahlenthemen eine wichtige Rolle. Je nach Eigenschaften eines Strahlenthemas, seiner gesundheitlichen Relevanz und dem gesellschaftlichen Umgang damit kommen unterschiedliche Ziele, Strategien und Instrumente der Risikokommunikation zum Einsatz. Neben zahlreichen Aktivitäten, die sich auf die Vermittlung von Informationen beziehen (Texte im Internet, Broschüren, Videoclips etc.), setzt das BfS auch den wechselseitigen Aspekt der Kommunikation durch entsprechende Formate um (Dialogveranstaltungen, Messen, Bürgertelefon, soziale Medien etc.). Der Umgang der Bevölkerung mit verschiedenen Strahlenthemen und die diesbezügliche Risikowahrnehmung werden anhand von sozialwissenschaftlichen Arbeiten im Rahmen von Forschungsprojekten erfasst (Berichte hierzu sind unter https://doris.bfs.de zu finden).

Im Folgenden werden 3 ausgewählte Felder der BfS-Risikokommunikation beschrieben, die einen Einblick in die Diversität der Risikokommunikation geben: aus dem Bereich der nichtionisierenden Strahlung die Themen *UV-Strahlung* sowie *hochfrequente und niederfrequente elektromagnetische Felder* und aus dem Bereich der ionisierenden Strahlung das Thema *Radon*.

### Risikokommunikation im Bereich UV-Strahlung

Der Bereich der nichtionisierenden Strahlung ist einer der Kommunikationsschwerpunkte des BfS und sehr vielfältig. Im Bereich der UV-Strahlung schützt sich eine des Hautkrebsrisikos grundsätzlich bewusste Bevölkerung nach wie vor nicht ausreichend vor der UV-Strahlung [5]. Gewohnheit und optimistischer Fehlschluss tragen zu den beobachtbaren Verhaltensweisen bei. Das Ziel der Risikokommunikation ist es hier also nicht primär, noch mehr Wissen zu vermitteln, sondern vielmehr weiterhin die gesamte Bevölkerung quer durch Altersgruppen und Bevölkerungsschichten darauf aufmerksam zu machen, dass das Risiko Hautkrebs jeden betrifft. Aufgeteilt in unterschiedliche Zielgruppen für die Risikokommunikation werden Schutzmotivation gefördert, Motive für Sonnenexpositionsverhalten hinterfragt und Barrieren für Schutzverhalten angesprochen. Zusätzlich wird hier mit einem Index für die sonnenbrandwirksame Bestrahlungsstärke – dem UV-Index – als Maßstab für angemessenes Sonnenschutzverhalten gearbeitet [6, 7]. Zahlreiche Akteure betreiben seit vielen Jahren aktive Kommunikation zum Schutz vor UV-Strahlung, um die Zahl der Hautkrebserkrankungen zu reduzieren. Ein gutes Beispiel für die Verstärkung der Risikokommunikation durch den Zusammenschluss verschiedener Akteure ist das fachübergreifende UV-Schutz-Bündnis, das seit dem Jahr 2012 unter Koordination des BfS für einen verantwortungsvollen Umgang mit UV-Strahlung eintritt [8].

### Risikokommunikation im Bereich der elektromagnetischen Felder des Mobilfunks und der Stromversorgung

Der Arbeitsschwerpunkt elektromagnetische Felder (EMF) stellt ein herausforderndes Spannungsfeld für die Risikokommunikation dar. Hier existieren für technische Anwendungen Grenzwerte, welche die Bevölkerung vor bekannten negativen Wirkungen elektromagnetischer Felder sicher schützen. Gegen Infrastruktureinrichtungen des Mobilfunks und der Stromversorgung wird jedoch vehement protestiert. Die wissenschaftliche Evidenz, dass die bestehenden Grenzwerte vor den wissenschaftlich nachgewiesenen gesundheitlichen Beeinträchtigungen schützen, steht einer konträr gelagerten Wahrnehmung (einer gesellschaftlichen Minderheit) möglicher Risiken durch die Exposition mit hoch- oder niederfrequenten elektromagnetischen Feldern gegenüber. Gefragt danach, was die Bevölkerung mit dem Begriff „Strahlung“ verbindet, nannte im Jahr 2019 knapp ein Viertel der Bevölkerung „Mobilfunk/Sendemasten/Handys/5G“ [5]. Aufgabe des BfS ist es nicht, Akzeptanz für Infrastrukturmaßnahmen, wie den 5G-Ausbau oder Stromnetzausbau, zu schaffen. Allerdings wird im gleichen Atemzug mit der Ablehnung der Infrastruktur auch die Sicherheit der Grenzwerte verneint und die wissenschaftliche Forschung abgelehnt, die zur Festlegung der Grenzwerte führt. Gerade in diesem Feld ist ein auffallender Bestandteil der Kommunikation daher die (Nicht‑)Verhandelbarkeit wissenschaftlicher Erkenntnisse. Neben protestierenden gibt es auch besorgte, verunsicherte und desinteressierte Bürger*innen. Ziele des BfS in diesem Bereich sind es, das Wissen der Bevölkerung über die Wirkungsweise und über mögliche (und unmögliche) Risiken elektromagnetischer Felder zu erhöhen, Verständnis für die wissenschaftlichen Sachverhalte zu stärken und Ansprechpartner in der Kommunikation zu sein.

### Risikokommunikation im Bereich ionisierende Strahlung, insbesondere Radon

Im Bereich der ionisierenden Strahlung liegt der aktuelle Schwerpunkt der Risikokommunikation auf der Umweltradioaktivität, insbesondere auf dem chemischen Element Radon, das weltweit nach dem Rauchen als einer der wichtigsten Faktoren für Lungenkrebs gilt. Ziel der Kommunikationsbemühungen ist es, Radon als Risikofaktor den Menschen bekannter zu machen, die potenzielle persönliche Betroffenheit zu verdeutlichen, die Anzahl an Radonmessungen und ggf. notwendigen Sanierungsmaßnahmen im privaten Umfeld zu steigern. In der Kommunikation werden dabei Querverweise zu anderen Themenbereichen gesucht, die bereits gesellschaftlich stärker verankert sind, wie zum Beispiel der Umgang mit Innenraumschadstoffen als Faktor der Innenraumlufthygiene. Trotz umfangreicher Risikokommunikationsbemühungen auf Basis langjähriger Erkenntnisse [9] bleiben sichtbare Kommunikationserfolge bisher aus. Die Kenntnis über Radon ist nach wie vor gering ebenso wie bisherige Messungen und Maßnahmen [5].

Zudem werden in der Information über ionisierende Strahlung grundlegende Kenntnisse über die Eigenschaften und Wirkung verschiedener Strahlungsarten vermittelt. Neben dem grundsätzlichen Anspruch, zu grundlegenden Kenntnissen über Radioaktivität in der Bevölkerung beizutragen, sollen damit auch die Grundlagen für die Krisenkommunikation im Falle eines radiologischen Notfalls gelegt werden. Die Herausforderung hierbei ist, das Augenmerk der Bevölkerung auf Themen zu legen, für die kein aktueller Handlungsbedarf besteht.

### Allgemeine Ziele und Inhalte der Risikokommunikation im Strahlenschutz

Anhand der genannten Beispiele lässt sich eine breite Spanne an Zielen und Inhalten der Risikokommunikation im Strahlenschutz feststellen:

- für ein Thema Aufmerksamkeit steigern,
- Wissenslücken schließen,
- eine angemessene Risikoeinschätzung stärken,
- die Kenntnis wissenschaftlicher und technischer Sachverhalte steigern,
- das persönliche Risiko verdeutlichen,
- das Wissen zum Umgang mit dem Risiko stärken,
- Schutzmotivation stärken,
- Handlungsbarrieren und -hemmnisse beseitigen.

Deutlich wird an diesen Beispielen, dass nicht nur die physikalischen Eigenschaften der Strahlung, sondern insbesondere auch die gesellschaftlich relevanten Eigenschaften der Strahlenart die Gestaltung der Risikokommunikation beeinflussen [10].

Es stellt sich nun die Frage, inwieweit sich diese Ziele der Risikokommunikation von der Krisenkommunikation abgrenzen lassen und inwieweit Risikokommunikation als der Krisenkommunikation vorgeschaltet bezeichnet werden kann.

## Herausforderungen für die Krisenkommunikation im radiologischen Notfallschutz

### Aufgaben der Krisenkommunikation

Der radiologische Notfallschutz stellt die Kommunikation über Strahlenrisiken vor besondere Herausforderungen: Die ohnehin komplexe wissenschaftlich-technische Sachlage der Strahlenrisiken wird in eine Krisensituation versetzt, in der die Aufnahme von Sachinformationen zunächst hinter die Bewältigung der Krisensituation tritt.

Die Ziele und Aufgaben der Krisenkommunikation sind vielfältig. Krisenkommunikation ist ein Teil des Schutzkonzeptes zur Bewältigung von Notfalllagen und soll dazu beitragen, dass die betroffene Bevölkerung empfohlene Schutz- und Verhaltensmaßnahmen umsetzt und damit die physische Gesundheit geschützt wird. Aufgabe der Krisenkommunikation ist es aber auch, psychosoziale Konsequenzen von Notfallsituationen zu verringern und so die psychische Gesundheit zu erhalten [11]. Dafür soll Krisenkommunikation die Sorgen der Bevölkerung adressieren, das Informationsbedürfnis von Menschen in einer Krise befriedigen und übermäßiger Angst vorbeugen. Mithilfe einer guten Krisenkommunikation erhalten Betroffene Hilfestellung, die Krisensituation besser zu bewältigen und die eigene Handlungsfähigkeit zu erhalten bzw. wiederherzustellen. Kommunikation soll zudem der Stigmatisierung von Personen und Gruppen in einem radiologischen Notfall entgegenwirken, aber auch die Krisenkommunikation selbst ist es, die Stigmatisierung in der Kommunikation vermeiden muss. Letztlich ist es auch Aufgabe der Krisenkommunikation, Fehlinformationen und Gerüchten entgegenzutreten [12].

### Eigenschaften guter Krisenkommunikation

Eine gute Krisenkommunikation muss zeitlich angemessen sein, Transparenz herstellen und die Zielgruppen erreichen. Dazu muss eine verständliche Sprache verwendet werden, die Kommunikation muss sich an den Bedürfnissen der Betroffenen orientieren und respektvoll gegenüber deren Situation sein. Zudem trägt das Prinzip „eine Botschaft – viele Stimmen“ („one message – many voices“) dazu bei, Informationen und Botschaften zwischen verschiedenen kommunizierenden Akteuren abzustimmen und dadurch das Vertrauen zu stärken. Denn Krisenkommunikation kann ihre Ziele nur erreichen, wenn die (betroffene) Bevölkerung Vertrauen in die zuständige(n) Behörde(n) hat. Vertrauen, dass Katastrophenschutzmaßnahmen sinnvoll sind, obwohl sie das soziale und ökonomische Leben der Betroffenen in diesem Moment auf den Kopf stellen. Vertrauen darauf, dass Entscheidungen unter Hinzuziehung allen verfügbaren Wissens getroffen werden. Vertrauen schafft Sicherheit und Schutzgefühl. Mangelndes Vertrauen trägt dazu bei, dass Gerüchte ihren Lauf nehmen und Fehlinformationen verbreitet werden. Vertrauen wird in Situationen mit hohem Stressfaktor eher davon beeinflusst, ob das Gegenüber zuhört, sich kümmert und Anteilnahme zeigt. Erst in zweiter Linie beeinflussen Kompetenz, Ehrlichkeit, Offenheit und weitere Faktoren (transparente, schnelle, klare und eindeutige Informationen) das Vertrauen in Institutionen und Behörden [13].

Eine besondere Herausforderung für die Krisenkommunikation stellt das geänderte Kommunikationsverhalten der Bevölkerung dar, das in den letzten Jahren zu beobachten ist. Für die öffentliche Meinungsbildung stehen schon längst nicht mehr hauptsächlich offizielle und über die klassischen Medien vermittelte Informationen zur Verfügung. Informationen finden ihre Verbreitung, Kommentierung, Abschwächung und Veränderung über soziale Medien. Die Nutzung von sozialen Medien durch die kommunizierenden Behörden selbst ist daher die logische Konsequenz, der seit einiger Zeit gefolgt wird. Allerdings bergen die unterschiedlichen Logiken einer Behördenkommunikation (u. a. sachlich, faktenorientiert, strukturiert) und der Social-Media-Kommunikation (z. B. emotional, einzelfallbezogen, kritisch und differenzierend) große Herausforderungen dafür, effizient an der Kommunikation über soziale Medien mitwirken zu können [14, 15].

### Veränderung der Krisenkommunikation im Verlauf der Phasen einer Krise

Ebenso, wie die Risikokommunikation die Brücke schlagen muss zwischen wissenschaftlicher Evidenz, der Risikowahrnehmung und dem Verhalten der Bevölkerung (siehe Abschnitt „Diversität der Risikokommunikation im Strahlenschutz“), ist es Aufgabe der Krisenkommunikation – unter höherem Zeitdruck und mit stärkerer Dringlichkeit – sich sowohl an den Ereignissen und den zu kommunizierenden Inhalten zu orientieren als auch die Situation der Betroffenen und ihre Reaktionen auf den Notfall, auf Informationen, Verhaltensempfehlungen und Einschränkungen zu berücksichtigen und die Kommunikation daran anzupassen.

Ein radiologischer Notfall wird in Phasen unterteilt, die sich unter anderem nach dem Status der Aktivitätsfreisetzung und der Art und Dringlichkeit der Maßnahmen richten [16]. Die Dringlichkeitsphase wird unterteilt in eine Vorfreisetzungsphase und eine Freisetzungsphase. Die Nachunfallphase wird unterteilt in eine Übergangsphase und eine langfristige Nachunfallphase.

Je nach Notfallphase ändern sich Inhalte, Zielgruppen, Botschaften und Kommunikationsformen der Krisenkommunikation und ebenso ändern sich der Anteil der Risiko- an der Krisenkommunikation und die Art der Risikokommunikation.

In der Dringlichkeitsphase steht die Bevölkerung unter Stress und ist zunächst nicht in der Lage, komplexe Informationen zu verarbeiten. In dieser Frühphase eines Ereignisses herrschen Schutzreaktionen sowie das Bedürfnis nach grober Einordnung der Ereignisse und der Bewertung der persönlichen Betroffenheit (inkl. nahestehender Personen) vor. Das vorrangige Bedürfnis der Menschen ist, den Schutz der eigenen Gesundheit und der Angehörigen sicherzustellen. Der Erhalt sozialer Bindungen und persönlicher Beziehungen ist für die meisten Menschen die wichtigste Grundlage für das persönliche Wohlbefinden und für den Umgang mit Stresssituationen. Je größer die wahrgenommene Betroffenheit, umso weniger prägt die bewusste (analysierende und abwägende) Auseinandersetzung mit den Ereignissen diese frühe Phase. Das Interesse an „Zahlen – Daten – Fakten“ ist zu diesem Zeitpunkt noch sehr gering. Die Menschen benötigen besonders in dieser Phase von Anfang an die Gewissheit, dass sich jemand engagiert und kompetent um die Eindämmung der Katastrophe kümmert und die Sorgen und Bedürfnisse der Bevölkerung ernst nimmt. Für die Kommunikation bedeutet dies vor allem in der Frühphase eines Ereignisses, Verhaltensempfehlungen und Handlungsanweisungen klar und eindeutig zu formulieren, sodass sie von der Bevölkerung leicht zu befolgen sind.

Erst in der Nachunfallphase eines radiologischen Notfalls, wenn sich die Ereignisse nicht mehr überschlagen, erhält die bewusste und kognitive Verarbeitung der Daten und Fakten einen höheren Stellenwert. Diese Phase kann einen langen Zeitraum umfassen, von der Übergangsphase, die je nach Unfallhergang bereits nach einigen Tagen eintreten kann, bis zur langfristigen Nachunfallphase, die bis zu Jahre und Jahrzehnte anhalten kann, wie man an den Beispielen der Reaktorunglücke von Tschernobyl 1986 und Fukushima 2011 sieht.^2^ In dieser Phase werden Informationen abgewogen, Zahlen und Daten werden wichtiger für die wahrgenommene Kontrolle der Situation. Die akute Krise ist vorbei, die Risikokommunikation erhält ein größeres Gewicht. Zudem spielen partizipative und dialogorientierte Formate der Kommunikation im Verlauf der Phasen eines Notfalls eine immer größere Rolle, da sie dazu beitragen, die Betroffenen im Umgang mit der Situation zu unterstützen.

Zu beachten ist, dass die Art der Informationsverarbeitung in Notfällen individuell unterschiedlich ist und eine genaue Zuordnung zu den Notfallphasen daher nicht möglich ist.

Einen wichtigen Einfluss auf die Informations- und Unterstützungsbedürfnisse haben die jeweilige Lebenssituation sowie Art und Ausmaß der Betroffenheit durch das Ereignis. So wurden zum Beispiel als besonders vulnerable Gruppen für psychische Beeinträchtigungen nach den Reaktorunfällen von Tschernobyl 1986 und Fukushima 2011 Mütter kleiner Kinder und die Aufräumarbeiter identifiziert [1]. In wirtschaftlicher Hinsicht betroffene Personen haben wiederum ein sehr spezifisches Bedürfnis nach Existenzsicherung.

## Abgrenzung und Gemeinsamkeiten von Risiko- und Krisenkommunikation

Risiko- und Krisenkommunikation findet innerhalb des gesamten Kreislaufs des Risiko- und Krisenmanagements statt [4]. In allen Phasen des Krisenmanagements spielen die Planung und der Einsatz von Risikokommunikation und Krisenkommunikation eine wichtige Rolle. Nach erfolgter Krisenbewältigung werden in der Phase der Auswertung und Evaluation alle gewonnenen Erfahrungen („lessons learned“) zusammengefasst und in die Vorbereitung auf die nächste Krisensituation einbezogen.

### Abgrenzung Risiko- und Krisenkommunikation

Im Kontext der Krisenkommunikation wird Risikokommunikation häufig gesehen als Information potenziell betroffener Bevölkerungsgruppen im Normalzustand, bevor es zu einem Notfall kommt. Risikokommunikation wird die Aufgabe zugeschrieben, durch Transparenz und Kommunikation Glaubwürdigkeit und Vertrauen zu fördern und in ruhigen Zeiten die Grundlage für eine gute Krisenkommunikation zu schaffen. Dazu gehören Informationen über die Grundlagen von Radioaktivität und die Wirkung von Strahlung auf den Menschen, über mögliche Auswirkungen radiologischer Notfälle, über Verantwortlichkeiten und Zuständigkeiten im Notfallmanagement und über Schutzmöglichkeiten. Ein Ziel der Risikokommunikation in diesem Kontext ist es, dass die Bevölkerung bereits im Vorfeld so viel Wissen zu dem Thema erhält, dass sie weiß, was im Notfall zu tun ist. In vielen Themenbereichen des Katastrophenschutzes kommt der Risikokommunikation im Rahmen der Prävention eine wichtige Rolle zu und kann dazu beitragen, das Eintreten von Krisen abzuschwächen und einen positiven Einfluss auf das Krisenmanagement auszuüben. Sie kann die Selbsthilfefähigkeit der Betroffenen im Vorfeld stärken. Wo eine direkte Information der Bevölkerung nicht funktioniert, weil das Interesse an dem Thema zu gering ist, kann Informationsmaterial vorbereitet und im Falle eines radiologischen Notfalls verwendet werden. Kommunikationskanäle sollten etabliert und beübt werden. Das Bewusstsein über die Bedürfnisse der Bevölkerung kann im Vorfeld gestärkt werden.

Die Grenzen dieses Ansatzes sind klar erkennbar. Wie schon die kurze Beschreibung der Risikokommunikationsaktivitäten in anderen Themenfeldern des Strahlenschutzes zeigen, ist es schwer, die Aufmerksamkeit für Themen zu gewinnen, für die keine aktuelle gesellschaftliche Relevanz besteht. Dies trifft auch auf den radiologischen Notfallschutz zu. Grundsätzlich werden Notfälle für die Öffentlichkeit erst dann relevant, wenn sie eintreten oder aber wenn deren (wiederkehrender) Eintritt so wahrscheinlich ist, dass entsprechende Vorbereitungen in den Alltag der Bevölkerung integriert werden, wie zum Beispiel bei der Vorbereitung auf Erdbeben in Japan. Unterstützend könnte auch eine Integration des Umgangs mit Risiken in den allgemeinen Bildungsbereich wirken, um die Befassung mit möglichen Krisensituationen und die Vorbereitung darauf präsenter und selbstverständlicher werden zu lassen.

Dennoch besteht durchaus Potenzial der Risikokommunikation im Allgemeinen, eine gute Grundlage für die Krisenkommunikation zu legen, da Risikokommunikation und Krisenkommunikation gemeinsame Anforderungen aufweisen [17].

### Gemeinsamkeiten von Risiko- und Krisenkommunikation

#### Arten der Kommunikation.

Insbesondere in der Risikokommunikation, aber auch in der Krisenkommunikation sind unterschiedliche Anteile an Care Communication (Fürsorgekommunikation) und der Consensus Communication (Konsenskommunikation) enthalten. *Care Communication* kommt zum Einsatz, wenn die wissenschaftliche Evidenz Anlass für Schutzkonzepte (z. B. UV-Strahlung) oder schützendes Verhalten (z. B. Radon) gibt. In vielen Fällen hinterfragt die Öffentlichkeit die wissenschaftliche Evidenz nicht, steht dem Risiko jedoch mit einer gewissen Apathie gegenüber (Nichtwissen, Nichthandeln). Ziel der Care Communication ist die Vermittlung des Risikos bzw. Schutzverhaltens an die betroffene Bevölkerung. Für eine erfolgreiche Kommunikation in solchen Bereichen sind Kenntnisse über den gesellschaftlichen Umgang mit dem Risiko erforderlich.

Die *Consensus Communication* tritt ein, wenn zu einem Thema (z. B. EMF: Mobilfunk, Stromnetzausbau) verschiedene Meinungen über die wissenschaftlichen Erkenntnisse im Raum stehen. In diesen Kommunikationsszenarien haben die ethisch-moralischen Wertvorstellungen der Beteiligten – Kommunikator, Bevölkerung – maßgeblichen Einfluss auf den Kommunikationsprozess. Die Zielgruppe drängt auf Mitbestimmung beim Umgang mit der Situation, daher sind dialogische Ansätze besonders wichtig. Anteile von Care und Consensus Communication finden sich auch in der Krisenkommunikation.

#### Die Rolle von Vertrauen.

Das Vertrauen darauf, dass der/die Kommunikator*in dieselben Werte vertritt und in Bezug auf das spezielle Thema eine hohe Kompetenz aufweist, spielt insbesondere in der Krisenkommunikation eine wichtige Rolle, wenn es darum geht, über behördliche Einschätzung zu informieren und behördliches Handeln und Empfehlungen zu vertreten. Je näher der/die Sprecher*in einer Behörde den Werten, Einstellungen und Sorgen der Bevölkerung ist, umso höher ist die Wahrscheinlichkeit, dass ihm/ihr vertraut wird. Vertrauen spielt aber auch in der Risikokommunikation eine wichtige Rolle und ist Bestandteil der Konzeptionen von Risikokommunikationsaktivitäten in verschiedenen Themenfeldern. Vertrauen besteht zwar themenspezifisch, dennoch können Risikokommunikationsaktivitäten in anderen Themenfeldern durchaus dazu beitragen, die Bekanntheit eines Akteurs zu steigern und seine Art, zu handeln und zu informieren, bekannt zu machen.

#### Kenntnis der Zielgruppe.

Das höchste Gut der Kommunikation ist die Kenntnis der Zielgruppe. Dies ist gültig für die Risiko- wie auch für die Krisenkommunikation. Um die Risikokommunikation im Kontext des gesellschaftlichen Umgangs mit einem Thema so wirkungsvoll wie möglich zu gestalten, gibt das BfS Untersuchungen in Auftrag, um die erforderlichen Kenntnisse über die Zielgruppen einzuholen. Leitend für die Fragestellungen sind dabei die Ziele der Kommunikation. Je spezifischer das Ziel der Risikokommunikation ist, umso spezifischere Informationen sind nötig über die Risikowahrnehmung der Bevölkerung, den Umgang mit Risikoinformationen, bisherige Verhaltensweisen, die Einstellung gegenüber Behörden sowie Schutzmotivationen und Handlungsbarrieren. Dazu kommen hilfreiche soziodemografische Informationen und Informationsquellen der Zielgruppen. Diese Erkenntnisse sind auch für die Krisenkommunikation von Nutzen. Erkenntnisse über die Bereitschaft der Bevölkerung, Handlungsempfehlungen zu folgen, haben gleichermaßen Relevanz in der Risiko- und Krisenkommunikation – wenngleich mit unterschiedlichem Gewicht, je nachdem welchen Anteil Care‑, Consensus- oder Krisenkommunikation haben.

#### Anpassung der Kommunikationsstrategie.

Erfahrungen mit der dynamischen Anpassung von Kommunikationsstrategien auf der Grundlage von Rückmeldungen aus der Bevölkerung zu Informations- und Kommunikationsformaten und Strategien des Risikomanagements sind ausschlaggebend für die erforderliche Flexibilität im Krisenfall. Risiko- und Krisenkommunikation haben gleichermaßen mit Fehlinformationen zu kämpfen. Wissens- und Informationslücken oder Unsicherheiten werden mit Gerüchten gefüllt. Geschichten, Emotionen und Einzelschicksale genießen eine hohe Aufmerksamkeit ebenso wie Widersprüche und von den allgemeinen Erwartungen Abweichendes. Erkenntnisse über Informationsquellen der Bevölkerung zu Strahlenthemen bieten wichtige Hinweise, mit welchen weiteren Ereignisdarstellungen und Bewertungen die Bevölkerung konfrontiert sein könnte. Dies ist gleichermaßen wichtig sowohl für die Risikokommunikation als auch für die Krisenkommunikation im radiologischen Notfall. Eine Analyse der anderen Akteure sowie von deren Botschaften und Inhalten zum fokussierten Thema legt den Grundstein für die Bildung von Kommunikationspartnerschaften und die Zusammenarbeit mit Multiplikator*innen.

## Schlussfolgerung

Risiko- und Krisenkommunikation haben unterschiedliche Anlässe und verschiedene Zielsetzungen. Allerdings ist die Kommunikation über Risiken immer auch – in variierendem Anteil – Bestandteil der Krisenkommunikation. Neben verschiedenen Zielsetzungen rund um den informierten Umgang mit Strahlenrisken kann Risikokommunikation zum Ziel haben, mithilfe bestimmter Kommunikationsformate und -inhalte Teile der Bevölkerung auf mögliche radiologische Notfälle vorzubereiten. Damit kann sie den Weg für einen leichteren Einstieg in die Krisenkommunikation ebnen. Da aber in ruhigen Zeiten die Aufmerksamkeit der Öffentlichkeit für Notfalllagen gering ist, ist die Wirkung derartiger Bemühungen für eine gute Krisenkommunikation eingeschränkt. Viel wichtiger ist es, in einer Krise den Prinzipien guter Krisenkommunikation zu folgen, wie sie im Abschnitt „Herausforderungen für die Krisenkommunikation im radiologischen Notfallschutz“ beschrieben sind. Für eine zeitnahe und angemessene Information der Bevölkerung in einer Krise ist es viel wert, wenn Informationsbausteine und grafisches Material bereits im Vorfeld erarbeitet wurden. Zahlreiche weitere wichtige Aspekte können und sollten kontinuierlich auch im Kontext anderer Risikokommunikationsthemen gelebt werden und damit eine gute Vorbereitung für die Krisenkommunikation sein: die Kenntnis des kommunizierenden Akteurs, die Bedeutung einer verständlichen Darstellung von Sachinformationen auch für Laien, die Rolle von Vertrauen, das Eingehen auf die Bedürfnisse der Zielgruppen, um physischen wie psychischen Schaden von ihnen abzuhalten, Respekt vor anderen Meinungen und Bewertungen sowie ein Verständnis für das Verhalten von Zielgruppen im Umgang mit Strahlung.

## Footnotes

1 Weitere Informationen zu den Aufgaben des BfS und zu den Themenbereichen sind auf der Homepage des BfS zu finden (www.bfs.de) und unter: https://www.bfs.de/DE/bfs/wir/aufgaben/aufgaben.html.

2 Entsprechende Informationen sind z. B. auf der Internetpräsenz der International Atomic Energy Agency www.iaea.org zu finden.
